# Supplementary material for: Increasing coverage and uptake of voluntary family planning in Uganda’s emerging municipalities and secondary cities: An implementation research study protocol
Source: PLoS One. 2024 May 10;19(5):e0293351. doi: 10.1371/journal.pone.0293351 (PMC11086862; doi:10.1371/journal.pone.0293351)
Supplement: S1 File — (DOCX) [file pone.0293351.s001.docx]

**Implementation risks and mitigation**

| **Risk** | **Mitigation** |
| --- | --- |
| 1. Incomplete and inconsistent data/information on FP measures that are of interest to the project. | - Gaps in data availability/quality will be addressed through targeted primary data collection. |
| 1. Lack of consensus among the diverse stakeholders | - Continuous engagement of stake holders throughout the project implementation. Meetings and workshops will be conducted to enlist Stakeholder support. |
| 1. The mismatch between stakeholder priorities and the project’s scope. | - Evidence-based criteria and clear guidelines will be used during the co-design workshops to provide systematic guidance and consistency to the process and convergence of ideas. In addition, the project’s scope and objectives as well as the rationale will be communicated to stakeholders. |
| 1. Potential resistance of providers or other stakeholders. | - Sensitization and awareness creation activities for providers (and the community) will be undertaken in collaboration with the city leadership at different levels, to obtain their buy-in and cooperation in the implementation of proposed activities. |
| 1. Inherent characteristics of the urban population such as high mobility, complexity and dynamism. These may affect the monitoring and reporting of project activities. | - As a mitigation strategy, we will assess the most feasible and realistic ways to monitor implementation and report outputs, taking into consideration the service delivery context. |
