# Supplementary material for: Increasing coverage and uptake of voluntary family planning in Uganda’s emerging municipalities and secondary cities: An implementation research study protocol
Source: PLoS One. 2024 May 10;19(5):e0293351. doi: 10.1371/journal.pone.0293351 (PMC11086862; doi:10.1371/journal.pone.0293351)
Supplement: Attachment — Submitted filename: response to the review-CB.docx [file pone.0293351.s002.docx]

A detailed point-by-point response to the Academic Editor’s comments and the way they are addressed in the paper is indicated below.

Journal Requirements:

When submitting your revision, we need you to address these additional requirements.

1. Please ensure that your manuscript meets PLOS ONE's style requirements, including those for file naming. The PLOS ONE style templates can be found at
   <https://journals.plos.org/plosone/s/file?id=wjVg/PLOSOne_formatting_sample_main_body.pdf> and 
   <https://journals.plos.org/plosone/s/file?id=ba62/PLOSOne_formatting_sample_title_authors_affiliations.pdf>

**Thank you for the suggestions. We have formatted the document following the provided guidelines.**

2. We note that the grant information you provided in the ‘Funding Information’ and ‘Financial Disclosure’ sections do not match.  When you resubmit, please ensure that you provide the correct grant numbers for the awards you received for your study in the ‘Funding Information’ section.

**Thank you for bringing this to our attention. We have duly reviewed the grant number and made the necessary corrections.**

3. Thank you for stating the following financial disclosure:
   "The implementation of the project is funded by John Templeton Foundation, Grant number 62045.  The funder had no role in the writing of this manuscript and therefore, identified error in writing and reporting is solely the responsibility of the authors. " Please state what role the funders took in the study. If the funders had no role, please state: ""The funders had no role in study design, data collection and analysis, decision to publish, or preparation of the manuscript.""  If this statement is not correct you must amend it as needed. 
   Please include this amended Role of Funder statement in your cover letter; we will change the online submission form on your behalf.

**Thank you for your suggestion. We have now included the funder's role in the required format as per your recommendation.**

4. We note that you have stated that you will provide repository information for your data at acceptance. Should your manuscript be accepted for publication, we will hold it until you provide the relevant accession numbers or DOIs necessary to access your data. If you wish to make changes to your Data Availability statement, please describe these changes in your cover letter and we will update your Data Availability statement to reflect the information you provide.

**We are still in the process of collecting and cleaning all the required data. This statement was intended for those who may want to use our data in future. We have amended the sentence.**

5. Please include a separate caption for each figure in your manuscript.

**These have been provided before reference list as:**

Figures

Fig 1: **Theoretical framework**

Fig 2: **Implementation theory of Change**

Fig 3: **Implementation phase**

Fig 4: **Indicators and their measurements**

**Supporting documents**

**S1: Implementation risks and mitigation**

Please review your reference list to ensure that it is complete and correct. If you have cited papers that have been retracted, please include the rationale for doing so in the manuscript text, or remove these references and replace them with relevant current references. Any changes to the reference list should be mentioned in the rebuttal letter that accompanies your revised manuscript. If you need to cite a retracted article, indicate the article’s retracted status in the References list and also include a citation and full reference for the retraction notice.

**References have been revised and they are correct**

**Additional Editor Comments:**

a) Specific aims should be reduced to three: As currently stated, Specific aim 2 is a repetition of aim 1 and aim 3 and can be incorporated into those two.

**We thank the academic editor for the suggestion. We have removed the objective 2.**

b) The use of both a conceptual framework and a theory of change is repetitive and may be confusing. For implementation, a theory of change may be more relevant. Authors could consider using only the theory of change and incorporating elements currently outlined in the conceptual framework into it.

**We agree with the academic editor that this was confusing. We have created a section on theoretical framework and have shown how this guided the development of the implementation theory of change.**

c) The use of so many frameworks in the same text is confusing for the reader. The authors should an overarching framework for the study: If the human centered design is the overarching framework, then Activities under Table 1 should be incorporated into Table 3 under the subheadings discover, ideation and prototype. For discover phase of the HCD, the data sources for the quantitative data reviews should be mentioned.

**As earlier mentioned, we have created a section on theoretical framework. We have left table 1 as it is since it would be important to have the research questions before the methods section. We think this arrangement aligns with the logical flow of information, enabling readers to understand the research objectives before delving into the methodology**

d) It is then not clear where the Replicating Effective Programs Framework fits into the study. if this is the main framework for the study, then the authors may consider not mentioning the human centered design.

**We thank the academic editor for this suggestion. We agree that this was confusing. Actually, the figure summarises the implementation phases and as such we have revised the text indicating how it aligns the HCD.**

e) Table 2 and its supporting text (Implementation risk mitigation) is important for programming but may not be critical to the research protocol and may be attached as an annex

**We thank the academic editor for pointing out this. We have made edits and have attached the table as a supplement.**

f) Authors may consider registering their protocol with a registering body and providing a registration number with the next submission.

**While our protocol cleared by the UNCST after comprehensives review and approval by Makerere University School of public health Institutional review board, we have considered the advice of having it registered on the Open Science Framework (OSF) repository Registries (https://osf.io/vqxu9 ; DOI: 10.17605/OSF.IO/VQXU9).**
